# The effects of sustained COVID‐19 emergency and restrictions on the mental health of subjects with serious mental illness: A prospective study

**DOI:** 10.1002/jcop.22886

**Published:** 2022-05-26

**Authors:** Annarita Barone, Martina Billeci, Sofia D'Amore, Michele De Prisco, Giuseppe De Simone, Eleonora Ermini, Vittorio Freda, Federica Iannotta, Adalgisa Luciani, Luca Pistone, Lorenza M. Rifici, Viviana M. Saia, Giancarlo Spennato, Francesco Subosco, Licia Vellucci, Giordano D'Urso, Diana Galletta, Michele Fornaro, Felice Iasevoli, Andrea de Bartolomeis

**Affiliations:** ^1^ Section of Psychiatry, Department of Neuroscience, School of Medicine University of Naples “Federico II” Naples Italy; ^2^ UNESCO Chair on Health Education & Sustainable Development at University of Naples “Federico II” School of Medicine Naples Italy

**Keywords:** anxiety, COVID‐19, mental health, perceived stress, serious mental illness, telepsychiatry, vaccines

## Abstract

Few longitudinal studies have so far investigated the impact of sustained COVID‐19 among people with pre‐existing psychiatric disorders. We conducted a prospective study involving people with serious mental illness (*n* = 114) and healthy controls (*n* = 41) to assess changes in the Perceived Stress Scale, Generalized Anxiety Disorder Scale, Patient Health Questionnaire, and Specific Psychotic Experiences Questionnaire scores 18 months after the COVID‐19 pandemic outset. Subjects underwent interviews with a mental health professional in April 2020 and at the end of the local third wave (October 2021). A significant increase in perceived stress was found in healthy controls, especially females. Psychiatric patients showed a significant worsening of anxiety symptoms compared to baseline records (*t* = −2.3, *p* = 0.036). Patients who rejected vaccination had significantly higher paranoia scores compared to those willing to get vaccinated (*U* = 649.5, *z* = −2.02, *p* = 0.04). These findings indicate that COVID‐19's sustained emergency may cause enduring consequences on mental health, soliciting further investigations.

## 1 INTRODUCTION

The recent COVID‐19 outbreak due to the severe acute respiratory syndrome coronavirus 2 (SARS‐CoV‐2) was declared a Public Health Emergency of International Concern on January 30, 2020 by the World Health Organization (2020) and subsequently a global pandemic on March 11.

In Italy, the first SARS‐CoV‐2 infection was officially documented on February 20, 2020 in Codogno, then the number of diagnosed cases increased exponentially during March and the first part of April (Micheli et al., 2021; January 30, 2020), resulting in 36%–50% excess deaths from any cause (Alicandro et al., 2020).

The Italian government quickly adopted extraordinary social distancing measures in the so‐called “red zones” in an attempt to contain and limit the spread of the infection, but was later forced to extend travel restrictions and ban on public gatherings to the entire country, placing roughly 60 million people in lockdown (“Decree of the President of the Council of Ministers 01 March 2020,” 2020). Nation‐wide lockdown and stay‐at‐home restrictions were unprecedented in recent world history, representing a drastic policy that, though considered necessary by most, has not been easily tolerated by everyone (McDowell et al., 2021). In addition, regions and autonomous provinces have been able to enact additional restrictive provisions, as in the case of Campania, the most populated region in South Italy.

Viral spread, mortality, national and local authorities' regulations, the initial lack of effective pharmacological interventions or vaccines affected global mental health. The general population has been overwhelmed by psychological distress, feelings of worry, anxiety, and discomfort (Ansari Ramandi et al., 2020; Demartini et al., 2020; Risal et al., 2020). In particular, perceived stress and coping mechanisms varied with gender differences (Graves et al., 2021; Tsukamoto et al., 2021), and female sex resulted to be associated with higher COVID‐19‐related perceived stress (Ali et al., 2021; Ochnik et al., 2021). A meta‐analysis of the longitudinal studies conducted in 2020 showed that the increase in mental health symptoms reported in March–April 2020 in the general population declined over time and was not significant in samples measured in May–June 2020 (Robinson et al., 2022). Nonetheless, further pandemic waves, the lengthening of the restrictions, and the exaggerated media exposure of COVID‐19‐related news (Torales, Barrios, et al., 2022) could have led to financial concerns, feelings of isolation, and therefore pronounced levels of chronic stress, depressive and anxiety symptoms.

In this context, the mental health conditions of vulnerable individuals (i.e., those already affected by a serious mental illness (SMI), namely schizophrenia, bipolar disorder, and major depressive disorder), represent a major concern (Gentile et al., 2020; Yao et al., 2020). Having a history of psychiatric illness has been regarded as one of the most relevant predictors of the negative psychological impact of quarantine (Brooks et al., 2020). Patients with SMI may be among the hardest hit subjects, as they may be more vulnerable to the COVID‐19 outbreak for a series of clinical and psychological factors (Druss, 2020). In the Italian context, the burden of the epidemic on psychiatric patients might have been increased by the discontinuation of outpatient health services, ordered by the authorities with the aim of concentrating the health resources on immediate COVID‐19‐related medical emergencies.

We were one of the first groups to describe the effects of forced restrictions on patients with severe psychiatric disorders, 2 months after the declaration of national lockdown in Italy. We previously reported that psychiatric patients were four times more likely to perceive high levels of COVID‐19‐related stress, and had significantly higher levels of anxiety and depressive symptoms compared to healthy controls at the start of the pandemic (Iasevoli et al., 2021).

Data from short‐term longitudinal studies have so far shown that the psychological impact of COVID‐19‐related confinement would not have been catastrophic on subjects with an established psychiatric diagnosis (Januel et al., 2021), and resilience attitude might initially have acted as a moderator that allowed adaptation to adversities during the first wave (Dejonckheere et al., 2021; Verdolini et al., 2021). Nonetheless, given the exceptional nature of circumstances created by the COVID‐19 pandemic, their persistence over time, and the unpredictability of their material and psychological consequences, long‐term monitoring of the mental well‐being of at‐risk individuals is paramount.

As an example, paranoid thoughts increase during long‐lasting crises due to terroristic attacks, natural disasters, or viral pandemics (van Prooijen & Douglas, 2017; Smallman, 2015). Paranoid subjects may be more likely to endorse conspiracy theories about pandemics, mask‐wearing, and vaccines, leading to risky behaviors and less adherence to public health countermeasures (Bronstein et al., 2021; Suthaharan et al., 2021; Tang et al., 2021).

In this scenario, we conducted a prospective study to assess the effects on the mental health of the prolonged state of emergency and restrictions. We investigated mental health trajectories in both psychiatric patients and nonpsychiatric subjects by re‐examining the severity of perceived stress, depressive, and anxiety symptoms at the end of the local “third wave” of COVID‐19 pandemic. In addition, we sought to analyze potential relationships between psychotic experiences of grandiosity or paranoia and willingness to vaccinate against the SARS‐CoV‐2.

## 2 MATERIALS AND METHODS

We assessed psychiatric patients and nonaffected controls after 1 month from the beginning of the first Italian lockdown (*t*0) (April 2020) (Iasevoli et al., 2021), and subsequently repeated the evaluation in the same subjects after the “reopening decrees” (*t*1) (October 2021), namely when the majority of cultural venues opened at 100% of capacity. Patients' evaluations were part of the procedures for remote monitoring and management of patients affected by SMI in the telehealth programs of the Psychiatric Unit of the University of Naples “Federico II.” Matched controls (age, sex, other major demographic variables) were recruited from Naples and surrounding areas by telephoning sequentially the phone numbers on a randomly selected page of the telephone directory.

We included in the prospective study patients who met the following criteria: (1) age 18–70 years; (2) Diagnostic and Statistical Manual of Mental Disorders (DSM‐5) diagnosis of schizophrenia or schizophrenia spectrum disorder, bipolar disorder, or major depressive disorder, recurrent episodes (American Psychiatric Association, 2013), as documented by clinical records; (3) clinical stability at the time of the assessments; (4) linguistic and cognitive capability of completing the questionnaires. The eligibility criteria for control subjects were: (1) age 18–70 years; (2) no history of previous or current psychiatric disorder; (3) linguistic and cognitive capability of completing the questionnaires. For both groups, exclusion criteria were: (1) being diagnosed with COVID‐19 or suspected of being infected at the time of the assessment or in the previous 6 months; (2) being hospitalized at the time of the assessment or suffering from a severe medical disease; (3) being a healthcare worker.

All procedures were approved by the Ethics Committee of the University School of Naples “Federico II” and were following the principles laid down by the Declaration of Helsinki, revised Hong Kong 1989. This study was registered with ClinicalTrials.gov (registration number: NCT04357769) on April 21, 2020. All participants provided consent to participate and were assured that their data would be published without identifying information. Patients were asked to complete a series of self‐rated questionnaires to explore symptoms related to the COVID‐19 outbreak and stay‐at‐home restrictions, including the 7‐item Generalized Anxiety Disorder Scale (GAD‐7) (Spitzer et al., 2006); the 10‐item Perceived Stress Scale (PSS) (S. Cohen et al., 1983); the Patient Health Questionnaire‐9 (PHQ‐9) for screening of depressive symptoms (Pidani et al., 2018); the Paranoia and Grandiosity subscales of the Specific Psychotic Experiences Questionnaire (SPEQ), which explore subthreshold psychotic symptoms (Ronald et al., 2014). The rating scales were read over the phone to participants or during telehealth visits by trained clinicians without providing comments that could introduce biases. For all participants, each evaluation referred to the previous 2 weeks. In addition, we collected demographic information such as age, sex, educational years, presence of medical disorders, socioeconomic, and vaccination status.

For clinical outcomes, the dichotomous variables were derived from the corresponding continuous ones, using standardized cut‐offs whenever available in the literature. The recommended cut‐off of ≥10 was applied as a diagnostic threshold for anxiety and depressive disorders (Kendrick et al., 2009; Spitzer et al., 2006). Although there is no predetermined cut‐off point for the PSS score, according to Cohen et al. subjects were classified into two categories, those with low stress (PSS ≤ 13) and those with clinically relevant perceived stress (PSS ≥ 14). On the basis of the frequencies (i.e., total counts) captured, we calculated the corresponding risk ratios with confidence intervals (CIs). Given the lack of reliable cut‐offs for grandiosity and paranoia, SPEQ scores were processed exclusively in terms of continuous variables.

The two‐way mixed analysis of variance (ANOVA) was used to detect any significant interaction between the independent variables time (the within‐subjects factor) and group (the between‐subjects factor) on the dependent psychopathological variables, namely GAD‐7, PSS, PHQ‐9, SPEQ‐P, and SPEQ‐G scores; the paired‐sample *t*‐test for repeated measures was used to detect significant changes over time in perceived stress, anxiety, depressive and psychotic symptoms in the two groups. Cohen's *d* was used to report effect size measures, whereby the values of 0.20, 0.50, and 0.80 represented small, medium, and large effect sizes, respectively (J. Cohen, 2013). For normally distributed variables, the independent‐sample *t*‐test was used to cross‐sectionally detect differences in perceived stress, anxiety, depressive and psychotic symptoms between patients and controls at both time points. For nonnormally‐distributed data the nonparametric Mann–Whitney *U* test was used for group comparisons.

Linear regression model and point biserial correlation were used to investigate whether sex and demographic variables were predictive of psychopathological outcomes. In all tests, significance was set at *p* < 0.05 (two‐tailed).

## 3 RESULTS

We initially recruited 205 patients and 205 controls in the first cross‐sectional evaluation (Iasevoli et al., 2021). One hundred and fourteen patients and 41 healthy controls were available and agreed to participate in the follow‐up assessment. Of the original respondents recruited in April 2020, 55.61% of patients and 20% of nonpsychiatric subjects participated again in October 2021. The final sample included 114 patients (*M*: 47.37%, mean age: 46.96 ± 13.16) and 41 healthy controls (*M*: 51.22%; mean age: 48.68 ± 13.51). Of 114 psychiatric patients, 67 were diagnosed with schizophrenia spectrum or other psychotic disorders, while 47 suffered from bipolar disorder or major depressive disorder, recurrent episode. No significant differences in mean age (independent *t*‐test, *p* > 0.05), gender rates, educational attainment, or presence of relevant medical disorders (*χ* ^2^, *p* > 0.05) have been found between patients and controls. Patients had lower economic status compared to healthy controls (*χ* ^2^, *p* < 0.05 *χ* = 33.29), frequently reporting to receive a disability pension (38.60%) or not being self‐sufficient (26.31%). Demographic data and mean rating scale scores are reported in Table 1. Rates of anxiety increased in psychiatric patients from 29.2% to 36.8% over the 18‐month period, whereas remained stable in controls (14.6%). The relative risk to suffer from anxiety symptoms at *t*1 in subjects with pre‐existing SMI compared to controls was 2.52 (95% CI: 1.16–5.48), the *z*‐statistic was 2.574 and the associated *p* = 0.01. Rates of clinically relevant perceived stress rose from 54% to 66.1% in patients and from 46.3% to 58.5% in the control group. The relative risk of experiencing moderate–severe perceived stress in subjects with a previous psychiatric diagnosis compared to those without a psychiatric history was 1.13 (95% CI: 0.84–1.51), *z* = 0.819, *p* = 0.41. Regarding depression rates, they remained fairly stable in psychiatric patients over time (38.9% at the *t*0, 38.4% at the *t*1) and decreased in non‐affected controls (17.1%–14.6%). The relative risk of psychiatric patients to suffer from a depressive disorder was 2.62 (95% CI: 1.21–5.70), *z* = 2.44, *p* = 0.015. Therefore, schizophrenia spectrum and mood disorder patients had a 2.5 times higher risk to suffer from anxiety or depression compared to healthy subjects at the end of the third wave (Table 2).

**Table 1:** The table describes means and SD for age and rates for nominal variables in psychiatric patients and nonaffected controls.

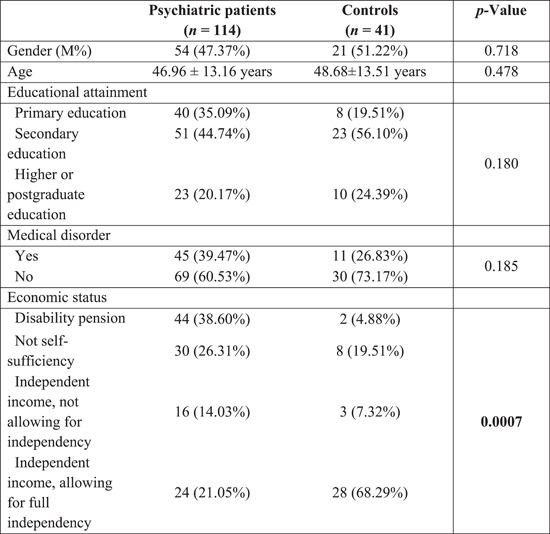

*Note*: Significant differences have been highlighted in bold.

**Table 2:** Rates of anxiety, depression, and moderate–severe perceived stress among the subjects enrolled in the study.

	Pre‐existing SMI		No pre‐existing SMI		RR		*p*‐Value	
	*t*0	*t*1	*t*0	*t*1	*t*0	*t*1	*t*0	*t*1
Anxiety	33 (29.2%)	42 (36.8%)	6 (14.6%)	6 (14.6%)	1.99	**2.52**	0.09	**0.01**
Depression	44 (38.9%)	7 (17.1%)	43 (38.4%)	6 (14.6%)	**2.28**	**2.62**	**0.02**	**0.01**
Moderate‐severe PS	61 (54%)	74 (66.1%)	19 (46.3%)	24 (58.5%)	1.16	1.13	0.42	0.41

*Note*: The table shows the risk ratio and corresponding *p* values.

Abbreviations: PS, perceived stress; RR, relative risk; SMI, severe mental illness.

We used the two‐way mixed ANOVA to examine potential differences in worsening of mental health symptoms between psychiatric and nonpsychiatric subjects from the first to the third pandemic wave. The model included interaction terms for timepoint (study enrollment and follow‐up assessment) and group (psychiatric subjects vs. controls).

No significant time‐group interaction on perceived stress, anxiety, depression, paranoia, and grandiosity symptoms was found (*p* > 0.05; see Table 3). The main effect of time showed a statistically significant difference in the mean PSS score at the different time points, *F* (1, 150) = 6.97, *p* = 0.009, partial *η* ^2^ = 0.04. The main effect of group showed that there was a statistically significant difference in mean GAD‐7 (*F* (1, 152) = 5.39, *p* = 0.022, partial *η* ^2^ = 0.03), PSS (*F* (1, 50) = 4.107, *p* = 0.044, partial *η* ^2^ = 0.03), PHQ‐9 (*F* (1, 150) = 16.54, *p* = 0.00008, partial *η* ^2^ = 0.10) and SPEQ‐P (*F* (1, 150) = 10.21, *p* = 0.002, partial *η* ^2^ = 0.06) between groups.

**Table 3:** The table shows the psychopathological measures recorded at both time points.

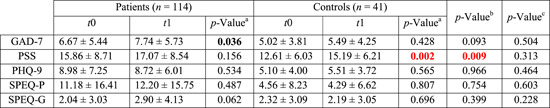

*Note*: Data are presented as mean values ± SD. Significant values are highlighted in bold. Significant values that survived after Bonferroni correction for multiple comparisons were given in red.

Abbreviations: GAD‐7, 7‐item Generalized Anxiety Disorder Scale; PHQ‐9, Patient Health Questionnaire‐9; PSS, Perceived Stress Scale; SPEQ‐G, Grandiosity subscales of the Specific Psychotic Experiences Questionnaire; SPEQ‐P, Paranoia subscales of the Specific Psychotic Experiences Questionnaire.

^a^Paired samples *t*‐test.

^b^Significance level for the hypothesis of no time effect.

^c^Significance level for the hypothesis of no time × group effect.

**Figure 1:**
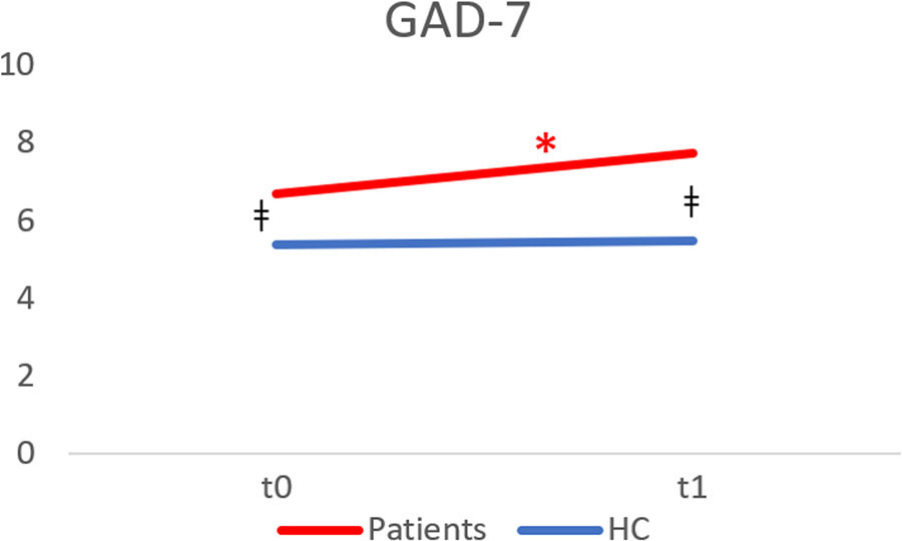
Line graph displaying GAD‐7 score trajectory over time in the two groups. The red asterisk indicates a significant increase in the patient group (*p* < 0.05) from *t*0 to *t*1. ǂ Indicates a significant difference between the groups (*p* < 0.05). GAD‐7, Generalized Anxiety Disorder Scale; HC, healthy controls.

Psychiatric patients showed a statistically significant increase in GAD‐7 score compared their baseline records (mean difference = −1.07; 95% CI: −2.07 to −0.73; *t*(112) = −2.3, *p* = 0.036, *d* = 0.19) (Table 3). After subdivision for macrodiagnostic groups, patients with mood disorders had significantly higher increase in GAD‐7 score compared to their baseline records (mean difference = −1.82; 95% CI: −3.20 to −0.45, *t*(45) = −2.68; *p* = 0.010) but this did not apply to psychotic patients. The difference in GAD‐7 score between patients and nonpsychiatric controls recorded at baseline (95% CI: 0.09–3.21; *t*(152) = 1.65, *p* = 0.038) remained significant in the follow‐up assessment (95% CI: 0.55–3.94; *t*(153) = 2.29, *p* = 0.010; Figure 1).

**Figure 2:**
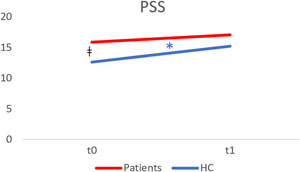
Line graph displaying PSS score trajectory over time in the two groups. The blue asterisk indicates a significant increase in the control group (*p* < 0.05) from *t*0 to *t*1. ǂ Indicates a significant difference between the groups (*p* < 0.05). HC, healthy controls; PS, Perceived Stress Scale.

Healthy control subjects showed a significant increase in PSS score (mean difference = −2.58; 95% CI: −4.19 to −0.98; *t*(40) = −3.26, *p* = 0.002, *d* = 0.42) over time (Table 2). The difference in PSS score between patients and healthy controls recorded at baseline (95% CI: 0.77–5.72; *t*(152) = 3.25, *p* =  0.011) was no longer detectable at the follow‐up assessment (95% CI: −0.63 to 4.38; *t*(151) = 1.87, *p* = 0.140). Therefore, the perceived stress levels in the two groups tend to be similar over time (Figure 2).

Since those who completed the follow‐up evaluation might have experienced different levels of anxiety and perceived stress from those who drop out of the study, we explored whether attrition depended on baseline variables and observed that both patients and controls who responded to the follow‐up evaluation did not differ significantly from nonrespondents in terms of PSS and GAD‐7 score at the baseline. Moreover, logistic binary regression revealed that neither the baseline PSS nor GAD‐7 scores were significant predictors of attrition.

**Figure 3:**
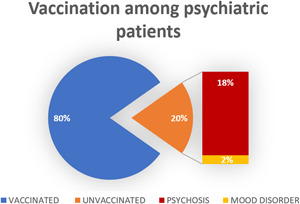
Pie charts showing the percentage of unvaccinated among psychiatric patients and the relative diagnoses.

In the patient population, 21 subjects (18.42%) were not vaccinated against SARS‐CoV2, despite Health Minister recommendations. Of interest, the most common diagnosis in this subset of patients was schizophrenia and psychotic spectrum disorders (90.48%; Figure 3). Among healthy subjects, only two were not vaccinated against SARS‐CoV2 (4.88%).

**Figure 4:**
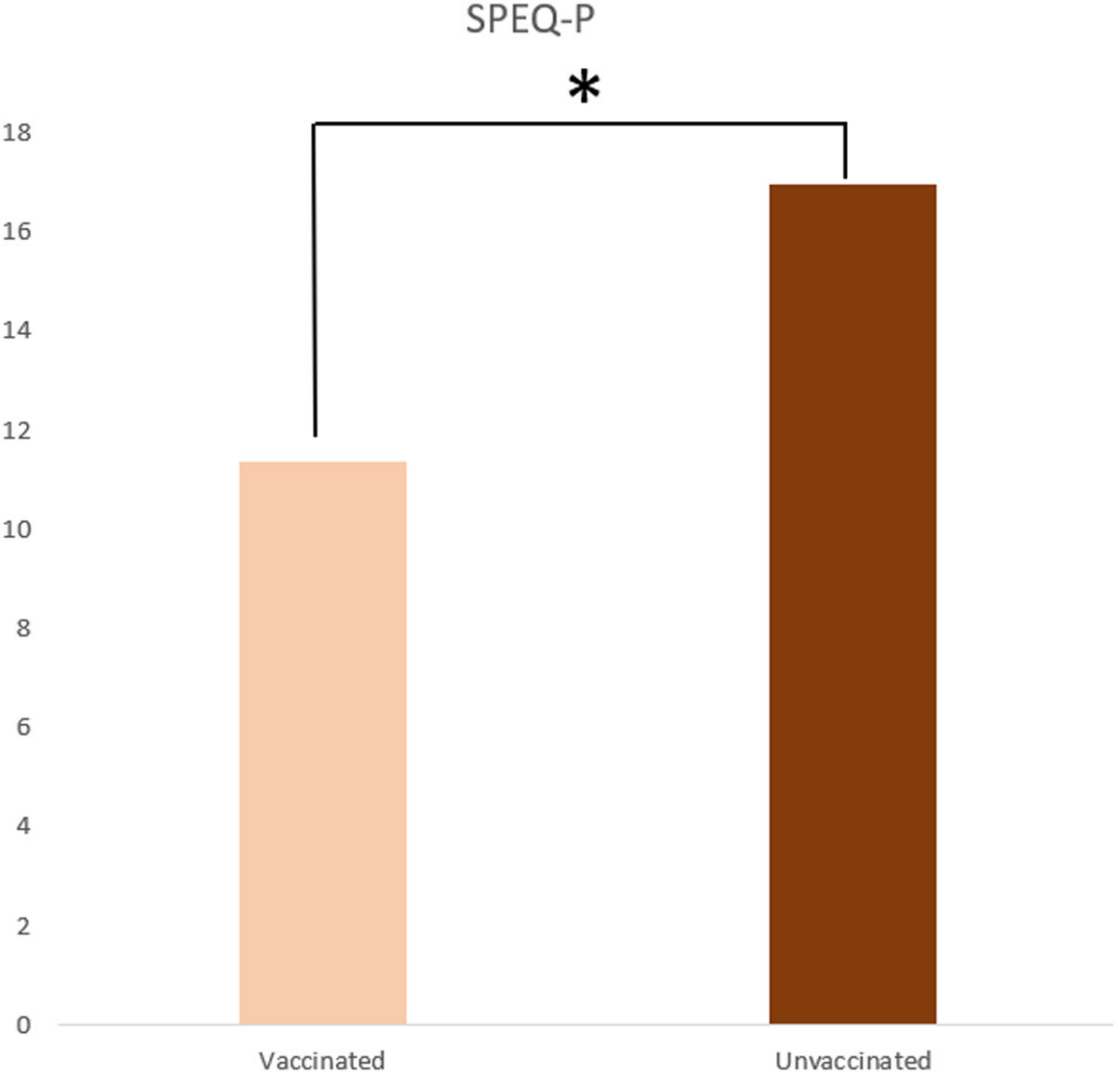
Among patients, unvaccinated ones exhibited significantly higher SPEQ‐P scores than those vaccinated. SPEQ‐P, Paranoia subscales of the Specific Psychotic Experiences Questionnaire.

We hypothesized that unvaccinated psychiatric patients may have a higher burden of psychotic symptoms compared to vaccinated ones. To evaluate potential differences in Paranoia SPEQ (SPEQ‐P) and Grandiosity SPEQ (SPEQ‐G) subscales between vaccinated and unvaccinated patients, we performed the nonparametric Mann–Whitney *U* test. Notably, unvaccinated psychiatric patients were found to have higher levels of paranoia as detected by the SPEQ‐P in comparison to vaccinated ones at the follow‐up (*U* = 649.5, *z* = −2.02, *p* = 0.043; Figure 4), whereas differences in SPEQ‐G between groups were not significant.

In the control group, females had significantly higher PSS levels both at baseline (95% CI: −8.71 to −1.79; *t*(39) = −5.252, *p* = 0.04) and follow‐up (95% CI: −8.95 to −1.81; *t*(39) = −5.378, *p* = 0.04) compared to males, but this did not apply to the group of patients. To determine the strength of correlation, a point‐biserial correlation between gender and PSS score was run in the healthy subject group, with males being coded as “1” and females as “2.” A statistically significant correlation of moderate to high magnitude was noticed at both timepoints (respectively, *r*_pb_ = 0.441, *p* = 0.004 and *r*_pb_ = 0.438, *p* = 0.04). The female sex accounted for 19.2% of the variation in the PSS score with *p* = 0.04, adjusted *R* ^2^ = 17.9%, a moderate effect size according to Cohen (2013) (J. Cohen, 2013).

## 4 DISCUSSION

The present study was aimed at investigating the effects of sustained COVID‐19 pandemic‐related restrictions on psychopathological variables in both SMI patients and non‐affected controls. There is conflicting literature describing how psychiatric patients, particularly those suffering from severe disorders, respond to overwhelming emergencies. Several studies exploring the effects of natural disasters, terroristic attacks, and critical political events (Bohlken & Priebe, 1991; Godleski et al., 1994; Koegler & Hicks, 1972; McMurray & Steiner, 2000; Stout & Knight, 1990) have reported seemingly unaffected or even improvement in their symptomatology. These reports disagree with the view that subjects affected by severe psychiatric illnesses represent a vulnerable population, at high risk for a further increase in psychiatric symptoms following stressful events (Jing & Katz, 2021).

Our findings highlight a significant negative impact of COVID‐19‐related restrictions on individuals' mental health. In summary, as related to the purported scopes of the work, we observed: (1) a slight but significant increase in anxiety levels in psychiatric patients compared to baseline; (2) a significant increase in perceived stress in nonpsychiatric subjects compared to baseline; (3) a significant association between psychological distress and female sex in the control group; (4) higher paranoia mean scores in vaccine‐hesitant patients compared to vaccinated patients.

The negative impact of the COVID‐19 pandemic on mental health was expected to be greater in people living with psychiatric disorders due to relatively lower social status, unhealthy lifestyles, and presence of comorbidities (Manuel et al., 2013; Plana‐Ripoll et al., 2019; Roy‐Byrne et al., 2009; Zolezzi et al., 2017). Accordingly, we detected an increase in anxiety symptomatology in the patient group, mainly attributable to the subgroup of patients suffering from mood disorders rather than psychosis. An impairment in novelty salience processing has been reported in patients suffering from psychotic disorders, for whom increased responses to neutral stimuli are counterbalanced by lower emotional responses to novel ones (Modinos et al., 2020). A reduced emotional response to pandemic‐related novelty contents may account for unaffected levels of anxiety in this subset of patients from the start of the pandemic. Furthermore, social isolation, loneliness, or exclusion may be a daily occurrence for psychotic patients who are often already socially isolated, have sedentary lifestyles, and commonly face stigma and discrimination from the general population (Michalska da Rocha et al., 2018; Pinto da Costa, 2020). An attenuated emotional response, on the one hand, a pre‐existing tendency towards social withdrawal on the other, could have, paradoxically, mitigated the effects of COVID‐19 emergency on this subset of patients, which although not showing a worsening of anxiety symptoms, might experience further social disengagement (Manchia et al., 2021). These findings are consistent with other research involving subjects with high‐functioning autism spectrum disorder, who have been reported to feel subjectively comfortable with social distancing measures and decrease in social interactions (Nisticò et al., 2022).

Contrary to our expectations, perceived stress levels are fairly stable over time in psychiatric patients. On the other hand, despite being relatively small in terms of sample size, the control group showed a significant increase in perceived stress over the three waves of the COVID‐19 pandemic. Unchanged levels of perceived stress in the patient group do not seem to underlie differences across diagnostic categories in facing the COVID‐19 pandemic, since the subgroup analysis for macrodiagnostic categories (i.e., mood disorder and psychosis) did not show significant results. These findings may rather suggest that subjects exposed to high rates of lifetime stressors are less prone to subjective report a worsening of symptoms (Fornaro et al., 2021), or that those who have access to mental health support in stressful situations tend to cope well (Kotlarska et al., 2021). In this respect, it should be emphasized that the telehealth service has ensured the continuity of mental health care protecting patients in our clinic as in many other facilities (Bashshur et al., 2016; Contreras et al., 2020; Hilty et al., 2013; Torales, Vilallba‐Arias, et al., 2022).

Moreover, our findings show that male and female subjects are differently burdened by COVID‐19‐related stressors. In fact, consistent with other studies (Graves et al., 2021; Ochnik et al., 2021; Tsukamoto et al., 2021), the female sex predicted higher levels of perceived stress in the control group, which should be taken into account when designing interventions aimed to address COVID‐19 pandemic‐related psychological issues.

Since several authors found an association between vaccine hesitancy and conspiracy beliefs (Bacon & Taylor, 2021; Bertin et al., 2020), we hypothesized that unvaccinated patients had a higher burden of paranoid symptoms in comparison to vaccinated ones. It is correct in our study, as unvaccinated patients had higher SPEQ‐P scores compared to vaccinated patients. Given the plasticity of delusional content concerning external events (Fischer et al., 2020; Stompe et al., 2003), the increased prevalence of jumping‐to‐conclusions bias (Zander‐Schellenberg et al., 2021), as well as the poorer awareness of health precautions (Kim et al., 2019), psychotic patients may be at greater risk of espousing sensationalistic interpretations of the COVID‐19 pandemic and embracing conspiracy theories about vaccines. Psychotic patients may suffer from several comorbidities such as hypertension, heart disease, and type II diabetes, which play an important role as risk factors for mortality in the SARS‐CoV2 infection (Bradley et al., 2021; Reyes et al., 2021). Therefore, this subset of individuals and their caregivers should be actively targeted by public and mental health prevention programs to mitigate the psychological COVID‐19 pandemic impact, as well as ameliorate the adherence to vaccination and recommended health precautions.

The main limitations of the study are the lack of a baseline evaluation before the COVID‐19 outbreak, and the attrition rate at the follow‐up, primarily affecting the control group. The most prevalent reason for drop‐out was no response to phone calls. While in the course of the first wave of pandemic the lockdown was complete and caused the suspension or reduction of most working activities allowing people to easily answer the home phone, many participants could no longer be found at home during the third wave, when restrictions have been relaxed and the activities were almost regularly resumed.

In conclusion, the data presented here demonstrated higher anxiety levels in SMI patients and outlined an increase in perceived stress also in non‐affected subjects over time, suggesting long‐lasting changes in global mental health due to the COVID‐19 state of emergency. These findings should be validated by larger sample sizes and future research should consider consequences in mental health as worthy of further investigations.

## 5 CONCLUSION

Confinement and restrictions have been associated with a worsening in global mental health from the early phase of the COVID‐19 outbreak. Patients living with SMI represent a vulnerable population at higher risk for depression, anxiety, and stress disorder symptoms. The results reported here confirm that anxiety symptoms worsened in psychiatric patients from April 2020 to October 2021. Perceived stress, depressive, and psychotic symptoms were stably severe in October 2021, although not worsened compared to April 2020. Paranoid thoughts were found higher in vaccine‐hesitant patients compared to those vaccinated.

## AUTHOR CONTRIBUTIONS

All authors contributed to the study conception and data collection. The first draft of the manuscript was written by Annarita Barone and Federica Iannotta, and all authors commented on previous versions of the manuscript. All authors read and approved the final manuscript.

## CONFLICTS OF INTEREST

The authors declare no conflicts of interest.

### PEER REVIEW

The peer review history for this article is available at https://publons.com/publon/10.1002/jcop.22886

## Data Availability

The data that support the findings of this study are available from the corresponding author upon reasonable request.
